# Short-term storage of alginate-encapsulated protocorm-like bodies of *Dendrobium nobile* Lindl.: an endangered medicinal orchid from North-east India

**DOI:** 10.1007/s13205-012-0090-4

**Published:** 2012-09-11

**Authors:** Padmaja Mohanty, Pynbeitsyon Nongkling, Meera C. Das, Suman Kumaria, Pramod Tandon

**Affiliations:** Plant Biotechnology Laboratory, Centre for Advanced Studies in Botany, North Eastern Hill University, Shillong, 793022 India

**Keywords:** *Dendrobium nobile*, Synthetic seed technology, Short term storage, Osmotica, Sucrose, Mannitol

## Abstract

Synthetic seed technology is an exciting and rapidly growing area of research as deals with conservation and storage of rare, endangered and desirable genotypes along with its easy handling and transportation. As propagation of many ornamental and medicinally important plant species is labour intensive, application of different growth retardants and osmotica in simple artificial seed system would dramatically reduce labour requirement by storing the germplasm in vitro. Moreover, the primary aim of developing in vitro storage methods is to reduce the frequent demands of subculturing and preserving the unique genetic constituent of the germplasm. *Dendrobium nobile* is a pharmaceutically important orchid mostly used in the Chinese herbal drug industry for its medicinal property. Commercial exploitation of this species has considerably depleted their population in wild. Hence, for conserving this valuable germplasm, short term in vitro storage of Protocorm-Like Bodies (PLBs) of *D. nobile* was carried out using different osmotica (sucrose and mannitol). It was observed that incorporation of low sucrose and mannitol (3 and 5 %) in the encapsulating matrix showed almost similar results with that of control. In all these cases, more than half of PLBs burst out from the matrix thus making these concentrations of sucrose and mannitol along with control not suitable for storage studies. However, with the increase in concentration to 7.5 and 12.5 % in the encapsulating matrix, no outburst of encapsulated PLBs was recorded till 60 days of storage; hence it can be concluded that these concentrations play an important role in minimizing the growth of PLBs during storage condition.

## Introduction

*Dendrobium nobile* Lindl. is a medicinally important epiphytic orchid and native to the states of North-east India, China, Myanmar, Thailand and Nepal. Attractive flowers and the pattern of flowering (large number of flowers per inflorescence) has made *D. nobile* commercially important in the cut flower market (Martin and Madassery 2006). Along with its ornamental importance it have been used in the Chinese herbal drug industry for its medicinal property (Ye et al. 2002). The stems of this species are used as a tonic to improve digestion and for promoting the production of body fluid (Anon 1999). However, anthropogenic pressures have rapidly decreased the natural habitat of this species with the consequent reduction in the number of plants. In the last two decades, in vitro techniques through micropropagation have played a major role in propagation and conservation of a large number of threatened plants (Dhar et al. 2000). However, somaclonal variations are often observed during serial subcultures (Withers 1991). To avoid such variations in vitro germplasm conservation strategy using in vitro storage technology has been developed.

Synthetic seed technology is an exciting and rapidly growing area of research as deals with in vitro conservation and storage of rare, endangered and desirable genotypes along with its easy handling and transportation (Kumaria and Tandon 2001; Germana et al. 2011). In vitro conservation involves the maintenance of explants in a pathogen-free environment for short - to medium- or long-term (Engelmann and Engels 2002). For short-term storage, the aim is to increase the interval between subcultures by reducing growth. Minimum growth condition for short- to medium-term storage can be followed in several ways, such as induction of osmotic stress with sucrose or mannitol (Wescott 1981), reduced temperature and/or light (Withers 1991) and incorporation of sub-lethal levels of growth retardant (Gupta 2001). Storage through slow growth methods is reproducible and widely applicable among different plant species and genotypes for conservation of germplasm (Withers 1991). Elite germplasms of various rare and endangered plant species like *Coffea Arabica* (Nassar 2003), *Rauvolfia tetraphylla* (Faisal et al. 2006), *Pterostylis saxicola* and *Diuris arenaria* (Sommrville et al. 2008), and *Pogostemon cablin* (Kumara Swamy et al. 2009) have been stored by in vitro methods using this slow growth technique. Roca et al. (1988) have successfully shown that nodal cuttings from meristem-derived plantlets of cassava (*Maniht esculentum*) could be maintained for 2 years on a medium with low osmotic concentration and activated charcoal. In Garlic (*Allium sativum*), the shoot tips could be stored for a period of 16 months following an increase in sucrose concentration to 10 % (El-Gizawy and Ford-Llyod 1987).

Though germplasm of many ornamental plants have also been successfully stored using this minimal growth technology, a very few reports have been made for orchids, viz. *Vanilla planifolia* (Divakaran et al. 2006), *Vanda coerulea* (Sarmah et al. 2010), *Cymbidium devonianum* (Das et al. 2011). Dubus (1980a, b) reported preservation of *Cymbidium* protocorms by increasing the sucrose concentration; however, many other authors have reported maintaining the cultures at low temperatures for storage and preservation (Sharma et al. 1992; Corrie and Tandon 1993; Datta et al. 1999; Das et al. 2008). Das et al. (2011) reported that in case of *C. devonianum* reduction in nutrient strength in the encapsulated matrix as well as low temperature increases the storage duration. However, the successful application of minimal growth technology requires the establishment of specific protocols for each type of explants and species under consideration (Watt et al. 2000). The aim of the present study is to develop an effective and applicable protocol for the short term in vitro storage of Protocorm-Like Bodies (PLBs) of *D. nobile* using different osmotica which is reported first time in case of *D. nobile.*

## Materials and methods

60-day-old PLBs of *D. nobile* were separated into single PLB and blot dried. The PLBs were then encapsulated in 3 % sodium alginate solution [dissolved in liquid MS medium containing different concentration of osmotica (sucrose and mannitol) in a range of 0.0–15.0 % (w/v)]. These were then singly dropped in 100 mM CaCl_2_·2H_2_O solution (also prepared by dissolving in liquid MS medium containing different concentration of sucrose and mannitol in the range as for sodium alginate). The alginate beads containing the PLBs were held for at least 15–20 min to achieve polymerization. The synthetic seeds obtained were taken out by decanting off the calcium chloride solution, washed with sterilized distilled water for 3–4 times, and surface dried with sterilized filter paper in Petri dishes for 5 min. Freshly prepared beads were then transferred in sterile Petri dishes and sealed with parafilm. Thirty synthetic seeds per Petri plate (three Petri plates for each treatment) were maintained and kept in dark at room temperature (25 ± 2 °C). In all the treatments, for storage studies, bursting of encapsulated beads was been recorded at intervals of 15 days and considered not suitable for storage. Only those unbursted beads were subjected to regeneration studies. Beads without containing any osmotica were considered as control.

In all the cases of regeneration studies, each Petri plate was taken out at a regular interval of 15 days and subcultured on regrowth medium [1/2 MS medium containing 2.0 % sucrose (w/v), 0.6 % agar (w/v) along with 1 mg/l BAP and 0.1 mg/l NAA, optimized media for PLB regeneration; Mohanty et al. 2012]. Survival percentage of stored synthetic beads after transferring to regrowth medium was recorded after 8 weeks of culture. The time taken by PLBs for emerging from beads, and for initiation of shoots and roots were recorded.

### Statistical analyses

The results were expressed as mean ± SE of three independent replicates of independent experiments. Data were subjected to analysis of variance (one way ANOVA) and Tukey’s multiple range tests using SPSS version 16.0.

## Results and discussion

**Fig. 1:**
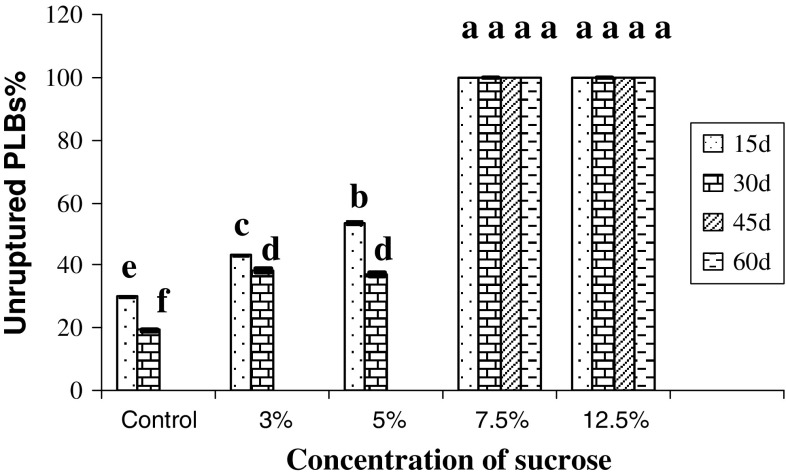
Effect of different concentrations of sucrose incorporated in MS medium in the encapsulating matrix on storage of *D. nobile* PLBs. Mean values having the same letter in each column are not significantly different at *P* < 0.05 (Tukey test) (*n* = 30)

**Fig. 2:**
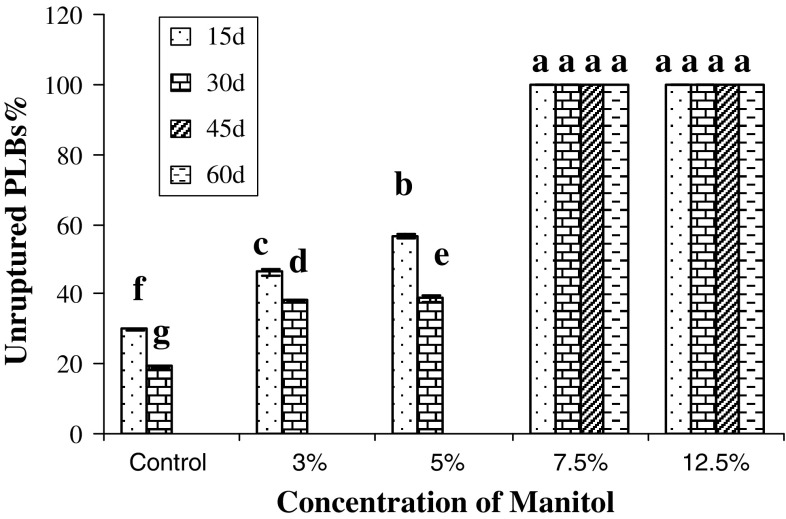
Effect of different concentrations of mannitol incorporated in MS medium in the encapsulating matrix on storage of *D. nobile* PLBs. Mean values having the same letter in each column are not significantly different at *P* < 0.05 (Tukey test) (*n* = 30)

**Fig. 3:**
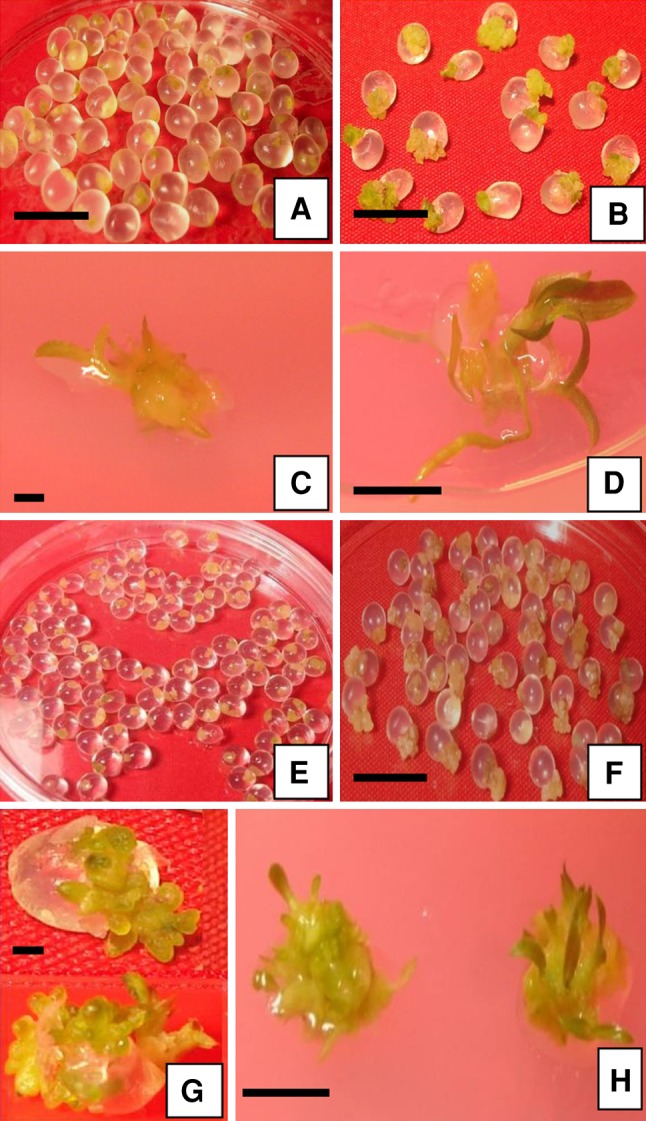
Plant regeneration from encapsulated PLBs of *Dendrobium nobile* after storage. **a** Encapsulated PLBs containing 7.5 % sucrose stored for 60 days (*bar* 1 cm). **b** Germinated encapsulated PLBs containing 7.5 % sucrose after 6 weeks of culture (*bar* 1 cm). **c** Shoot initiation from encapsulated PLBs containing 7.5 % sucrose after 10 weeks of culture (*bar* 1 mm). **d** Root initiation after 15 weeks of culture (*bar* 1 cm). **e** Encapsulated PLBs containing 7.5 % mannitol stored for 60 days. **f** Emergence of encapsulated PLBs after 30-day storage at 3 % mannitol (*bar* 1 cm). **g**, **h** Shoot initiation from encapsulated PLBs containing 7.5 and 12.5 % mannitol after 9 and 11 weeks of culture respectively (*bar* 1 mm and 1 cm, respectively)

Incorporation of sucrose and mannitol in the encapsulated matrix does affect the storage condition of the beads. Figures 1 and 2 show the effect of different concentration of sucrose and mannitol in the encapsulating matrix on storage of PLBs. In all these cases, more than half of PLBs burst out from the matrix in case of 3 and 5 % of sucrose and mannitol along with control, thus making these concentrations not suitable for storage studies. However, with increase in concentration to 7.5 and 12.5 % in the encapsulating matrix, no outburst of encapsulated PLBs was recorded (Fig 3a, e) till 60 days, hence it can be concluded that these concentrations play an important role in minimizing the growth of PLBs during storage condition. This may be due to osmotic stress imposed on the PLBs by higher concentration of sucrose and mannitol. Increased osmotic stress has been associated with cell plasmolysis resulting in slower cell division (Loveys et al. 1975) and cell growth inhibition (Wong and Sussex 1980). PLBs encapsulated in the beads containing 15 % sucrose and mannitol in the encapsulating matrix died after 10 days which may be due to increased rate of cell dehydration resulting in cell death. Therefore, the regeneration studies of only these concentrations (7.5 and 12.5 %) of sucrose and mannitol were further carried out.

On regeneration medium highest survival percentage (78.20 ± 0.41) of stored encapsulated PLBs was obtained when 7.5 % mannitol was incorporated in the matrix followed by 64.56 ± 0.38 % with 7.5 % sucrose (Table 1). But with increase in mannitol as well as sucrose concentration to 12.5 % in the encapsulating matrix, a decrease in survival percentage (60.00 ± 0.00 and 54.13 ± 0.12 %, respectively) of stored encapsulated PLBs was recorded. Similarly, emergence of PLBs from the beads was much faster when 7.5 % sucrose was incorporated (Fig. 3b) whereas with increase in sucrose concentration to 12.5 % there was a delay in emergence of PLBs when cultured on the regeneration medium. Normal shooting and rooting were observed in encapsulated PLBs incorporated with 7.5 % sucrose (Fig. 3c, d) whereas shoot and root development were completely inhibited when 12.5 % sucrose was incorporated in the encapsulating matrix. A nearly similar result was obtained in case of mannitol. The time taken in shoot and root development was much faster in PLBs encapsulated with 7.5 % mannitol as compared to PLBs encapsulated with 12.5 % mannitol (Table 1; Fig. 3g, h). It has been reported by many workers that relatively higher concentration of sucrose in the alginate matrix significantly decreased plant development, especially root formation. High levels of sucrose have been found to have adverse effects on shoot and root morphogenesis (George 1993; Panis 1995), which was also observed in our result. The sugar alcohol mannitol is most widely employed as pregrowth media additive for preservation studies (Withers and King 1980; Ng and Hahn 1985). However, variations exist between plants in their physiological and structural responses to osmotically active compounds. Pritchard et al. (1986) reported growth rate reduction and cell wall thinning in Sycamore and Soybean cells following osmotic stress due to higher concentration of mannitol but emphasized that cells display a different capacity for osmotic adjustment and alteration in their cytoplasmic component. Dehydration of PLBs at higher concentrations of mannitol results in the suspension of cell division and growth. Espinoza et al. (1986) have also reported that mannitol in the medium exerts an osmotic stress that leads to reduction in growth rate, hence acts as an effective osmoticum for short-term storage.

**Table 1:** Effect of different concentrations of sucrose and mannitol incorporated in MS medium in the encapsulating matrix on regeneration of *D. nobile* PLBs, cultured on regeneration medium (1/2 MS  + 1 mg/l BAP + 0.1 mg/l NAA) stored for 60 days

Treatments (%)	Regeneration % (recorded on 8th week)	Time taken for regeneration (weeks)		
		I	II	III
Sucrose				
7.5	64.56 ± 0.38^b^	5	10	15
12.5	54.13 ± 0.12^d^	7	–	–
Mannitol				
7.5	78.20 ± 0.41^a^	6	9	14
12.5	60.00 ± 0.11^c^	8	11	16

Mean values having the same letter in each column are not significantly different at *P* < 0.05 (Tukey test) (*n* = 15)

*Stage I* Emergence of PLBs from beads, *Stage II* Shoot initiation, *Stage III* Root initiation, – no regeneration

## Conclusion

In conclusion, this study developed highly effective techniques for synthetic seed production, short-term storage and distribution of *D. nobile* germplasm. The present work is first of its kind in case of short-term storage of PLBs using sucrose and mannitol as osmotica. Hundred percent of encapsulated PLBs of *D. nobile* could be stored till 60 days using sucrose (7.5 and 12.5 %) and mannitol (7.5 and 12.5 %). But among the all, 7.5 % mannitol was proved to be the best osmoticum for the storage. Following the protocol or with a little modification, conservation and storage of many rare, endangered and threatened orchid species will be possible.
